# The distinguishing NS5-M114V mutation in American Zika virus isolates has negligible impacts on virus replication and transmission potential

**DOI:** 10.1371/journal.pntd.0010426

**Published:** 2022-05-10

**Authors:** Nias Y. G. Peng, Alberto A. Amarilla, Leon E. Hugo, Naphak Modhiran, Julian D. J. Sng, Andrii Slonchak, Daniel Watterson, Yin Xiang Setoh, Alexander A. Khromykh

**Affiliations:** 1 School of Chemistry and Molecular Biosciences, The University of Queensland, St. Lucia, Queensland, Australia; 2 Mosquito Control Laboratory, QIMR Berghofer Medical Research Institute, Herston, Queensland, Australia; 3 Australian Infectious Diseases Research Centre, Global Virus Network Centre of Excellence, Queensland, Brisbane, Australia; WRAIR, UNITED STATES

## Abstract

During 2015–2016, outbreaks of Zika virus (ZIKV) occurred in Southeast Asia and the Americas. Most ZIKV infections in humans are asymptomatic, while clinical manifestation is usually a self-limiting febrile disease with maculopapular rash. However, ZIKV is capable of inducing a range of severe neurological complications collectively described as congenital Zika syndrome (CZS). Notably, the scale and magnitude of outbreaks in Southeast Asia were significantly smaller compared to those in the Americas. Sequence comparison between epidemic-associated ZIKV strains from Southeast Asia with those from the Americas revealed a methionine to valine substitution at residue position 114 of the NS5 protein (NS5-M114V) in all the American isolates. Using an American isolate of ZIKV (Natal), we investigated the impact of NS5-M114V mutation on virus replication in cells, virulence in interferon (IFN) α/β receptor knockout (*Ifnar*^-/-^) mice, as well as replication and transmission potential in *Aedes aegypti* mosquitoes. We demonstrated that NS5-M114V mutation had insignificant effect on ZIKV replication efficiency in cells, its ability to degrade STAT2, and virulence *in vivo*, albeit viremia was slightly prolonged in mice. Furthermore, NS5-M114V mutation decreased mosquito infection and dissemination rates but had no effect on virus secretion into the saliva. Taken together, our findings support the notion that NS5-M114V mutation is unlikely to be a major determinant for virus replication and transmission potential.

## Author summary

Zika virus (ZIKV) emerged to cause outbreaks in Southeast Asia and the Americas during 2015–2016. However, the scale of outbreaks in Southeast Asia were significantly smaller compared to epidemic in the Americas. A methionine to valine amino acid mutation at residue position 114 of the NS5 protein (NS5-M114V) is hypothesized to influence the epidemic outcomes of ZIKV, which led to the large-scale epidemic that occurred in the Americas. By analyzing infection of mammalian and mosquito cells, IFNα/β receptor knockout (*Ifnar*^-/-^) mice and *Aedes aegypti* mosquitoes with engineered ZIKV isolates containing either methionine or valine at residue position 114 of the NS5 protein, we demonstrated that the NS5-M114V mutation did not affect virus replication efficiency and STAT2 degradation in cells, virulence in mice, or virus secretion into the mosquito saliva. The NS5-M114V mutation slightly prolonged viremia in *Ifnar*^-/-^ mice and reduced mosquito infection rate. Collectively, our findings suggest that the NS5-M114V mutation is unlikely to have significantly influenced the ZIKV epidemic in the Americas.

## Introduction

First discovered in Uganda in 1947 [1], Zika virus (ZIKV) came into prominence when it began to cause large outbreaks as well as severe neurological complications termed congenital Zika syndrome (CZS) [2–5] throughout the Pacific Islands and Latin-America from 2007–2016 [6–10]. ZIKV encodes a ~11kb RNA genome consisting of 5’ and 3’ untranslated regions, with a single polyprotein that encodes for three structural and seven nonstructural viral proteins; namely capsid (C), pre-membrane (prM), envelope (E), nonstructural proteins 1 (NS1), NS2A, NS2B, NS3, NS4A, NS4B, and NS5 which facilitates the virus life cycle and antagonism of host innate immune responses [11,12].

All ZIKV strains prior to the epidemic in Latin-America are phylogenetically grouped into either the African- or Asian-lineages [13]. ZIKV strains isolated from the Latin-America epidemic were found to have originated from the Asian-lineage and were subcategorized into an American subclade within the Asian-lineage [14]. Several adaptive mutations and/or mutational reversions identified from phylogenetic analyses between the Asian-lineage and American subclade such as prM-S17N [15], C-T106A [16], E-Q350L/T397S/V473M [16,17], NS1-A188V [18,19] and NS5-M872V [16], either alone or in combination [20], were shown to contribute to the ZIKV emergence in the Pacific and Americas by enhancing virus fitness, neurovirulence and host-vector transmissibility [21–23].

**Fig 1:**
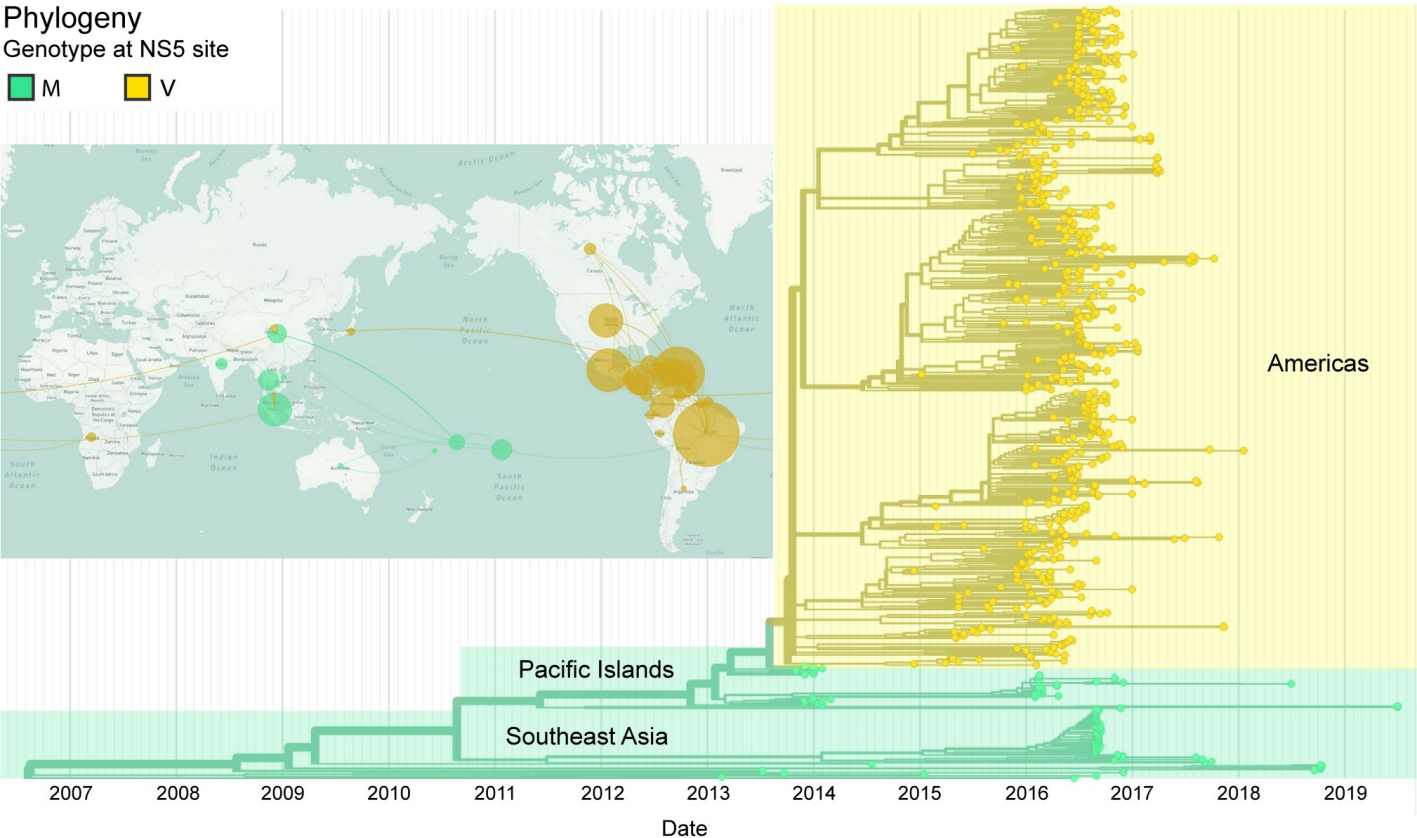
Phylogeographic analysis of the NS5-M114V mutation during the epidemiological spread of ZIKV from Asia to the Pacific Islands and Americas. This analysis was constructed using Nextstrain (https://nextstrain.org/zika, with permission from Trevor Bedford) with the use of 667 ZIKV genomes globally sampled from February 2013 to July 2019 (accurate at the time of writing) [27]. The geographic map denotes regions where reported cases of ZIKV outbreaks occurred from 2007 to 2019 (highlighted in colored circles). Infections caused by ZIKV strains containing the pre-American outbreak amino acid methionine at residue position 114 (M114) are colored in teal while ZIKV strains containing the American outbreak amino acid valine at the residue position (V114) are colored in yellow. Respective lines connecting respective highlighted circles depict the spread of NS5-M114V mutation during its global emergence. The size of the highlighted circles is relative to the magnitude of outbreaks. The phylogeny of ZIKV isolates globally sampled is color-coded similarly to the geographic map to differentiate the presence of either M114 or V114 among the isolates. As observed, all American outbreak ZIKV strains contained valine at residue position 114 of NS5 protein. The geographic map data constructed using Nextstrain was derived from OpenStreetMap (https://www.openstreetmap.org/#map=2/33.3/143.1), made available under the Open Database License (https://www.openstreetmap.org/copyright) and used under a CC-BY-4.0 license.

Most strikingly, a methionine (M) to valine (V) amino acid mutation at residue position 114 (M114V) located in the methyltransferase (MTase) domain of the NS5 protein distinguishes the isolates of the Americas from Asia [21–23]. The M114V mutation in NS5 was also absent in the smaller outbreaks in Yap Island and French Polynesian in 2007 and 2013, respectively; and only emerged in the Brazilian/American strains at the start of the larger 2015–2016 outbreak [21–23]. Importantly, M114V mutation is absent in strains circulating in Southeast Asia during the same period–which caused relatively smaller outbreaks compared to the one in the Americas [24–26] (Fig 1), which led us to hypothesize that NS5-M114V mutation may be an important determinant in the outbreak potential of ZIKV.

A previous study did not find a significant effect of NS5-M114V change on ZIKV replication in cells as well as pathogenesis and virulence in interferon receptor alpha/beta and gamma deficient (*Ifnar-*α/β/γ^-/-^) AG6 mice [28]. However, the impact of this mutation on virus virulence in the *Ifnar-*α/β^-/-^ mice that remain competent in IFNγ response and transmission potential in *Ae*. *aegypti* was not investigated. Herein, we engineered a NS5-V114M mutation into an American-lineage ZIKV-Natal isolate using our established circular polymerase extension reaction-based reverse genetics system [29]. This mutant (M114) was then compared to the wildtype ZIKV-Natal isolate (V114) for replication in various cell lines, virulence in *Ifnar*^-/-^ (*Ifnar-*α/β^-/-^) mice [30], and transmission potential by *Aedes aegypti* (*Ae*. *aegypti*) mosquitoes. Contrary to our hypothesis, we found that the NS5-M114V mutation did not enhance ZIKV replication or STAT2 degradation *in vitro*, and it did not increase virulence in *Ifnar*^-/-^ mice, albeit slightly prolonged the period of viremia. NS5-M114V mutation also decreased infection (bodies) and dissemination (legs and wings) rates in *Ae*. *aegypti* mosquitoes but had no effect on transmission potential (secretion into saliva).

## Materials and methods

### Ethics statement

Mouse infections were conducted under approval from the University of Queensland Animal Ethics Committee (AEC approval SCMB/010/19).

### Cell lines

African green monkey kidney cell line clone Vero76 (ATCC; CRL-1587), adenocarcinoma human alveolar basal epithelial A549 cell (ATCC; CCL-185), and interferon–alpha/beta receptor deficient immortalized murine embryonic fibroblast (*Ifnar*^-/-^ MEF) cell lines were cultured in DMEM (Gibco) at 37°C with 5% CO_2_. *Ae*. *albopictus* C6/36 (ATCC; CRL-1660) and *Ae*. *aegypti* Aag2 cells (RRID: CVCL_Z617) were cultured respectively in RPMI (Gibco) and a 1:1 (v/v) mixture of Schneider’s Drosophila medium and Mitsuhashi & Maramorosch medium (Sigma, USA) at 28°C with 5% CO_2_. All culture media were fortified with 10% (v/v) fetal bovine serum (FBS), 2 mM L-glutamine, 100 Units/mL penicillin, 100 μg/mL streptomycin. All cell lines were regularly tested and remain free from mycoplasma contamination in this study.

### Generation of ZIKV-Natal V114M mutant virus

**Fig 2:**
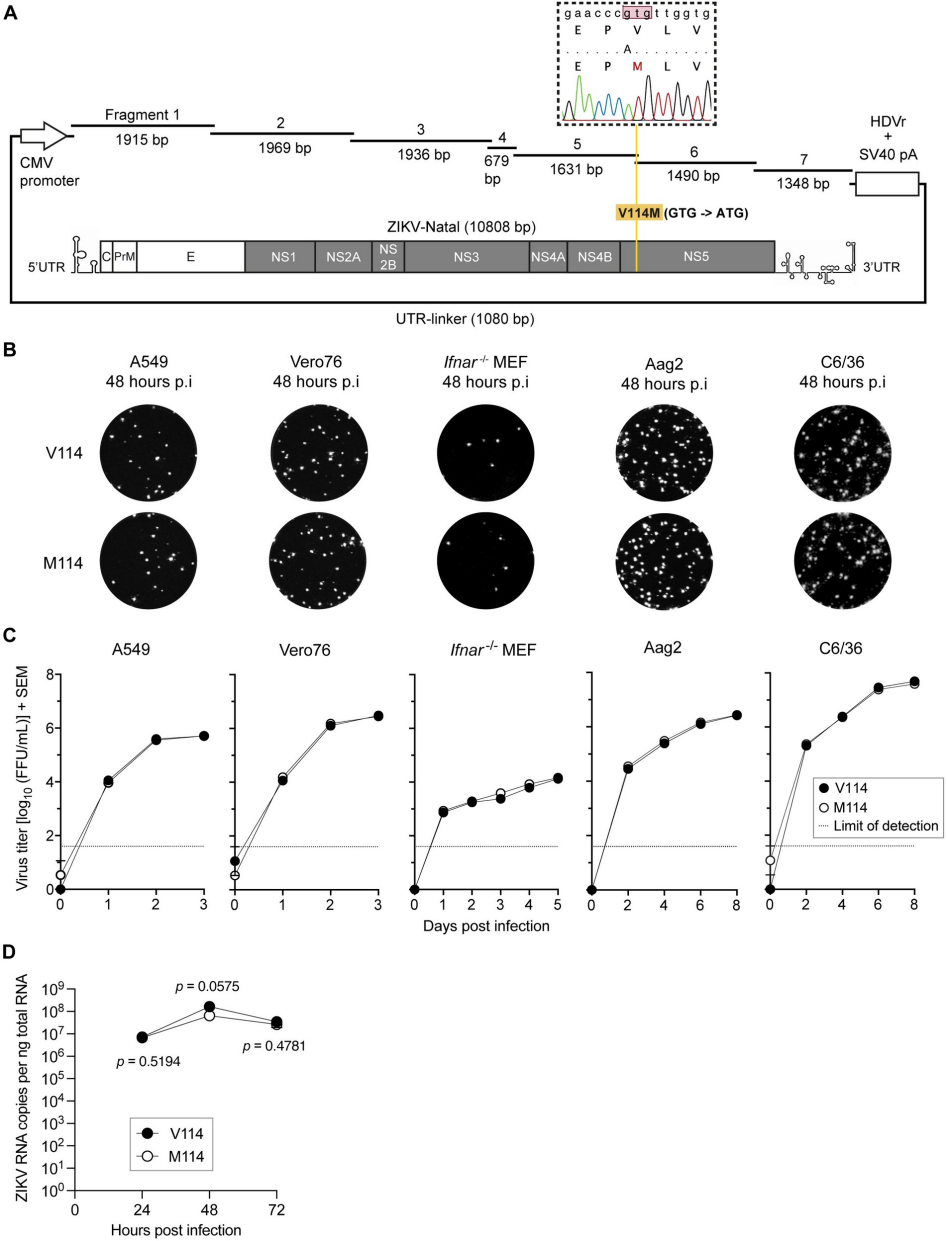
Generation of ZIKV NS5-V114M mutant (M114) based on ZIKV-Natal strain by CPER and its characterization *in vitro* [29]. (**A**) Schematic representation of the NS5-V114M mutation in the ZIKV-Natal genome and verification of the NS5-V114M mutation in CPER-recovered virus by Sanger sequencing. (**B**) Representative foci morphologies of indicated cells infected with either V114 or M114 ZIKV strains at 48 hours post-infection (hpi) as determined by immuno-plaque (iPA) assay on Vero76 cells. (**C**) Comparison of growth kinetics between V114 and M114 ZIKV strains in representative vertebrate and invertebrate cells. Cells were infected at MOI 0.1 and incubated for 3 days (vertebrate) or 8 days (invertebrate). Virus titers shown are log_10_ transformed mean values and represented as FFU/mL ± SEM, as determined by iPA on Vero76 cells; n = 3 independent experiments. Dotted lines represent limit of detection at 1.6 log_10_ FFU/mL. Significant differences between the means were determined using two-way ANOVA with Tukey’s multiple comparison test where *p* ≤ 0.05 (95.0% CI); “ns”, not significant, *p ≤ 0.05, **p ≤ 0.005, ***p ≤ 0.0005, ****p ≤ 0.0001. (**D**) Viral RNA replication in A549 cells infected with MOI 0.1 of V114 or M114 was assessed at indicated time points for 72 hpi. Significant differences between the means were determined using two-way ANOVA with Fisher’s LSD test for multiple comparisons where *p* ≤ 0.05 (95.0% CI).

The wildtype and V114M reverse mutant ZIKV-Natal (Genbank accession number KU527068.1) strains were generated using circular polymerase extension reaction (CPER) as previously described [29]. To facilitate a valine (V) to methionine (M) substitution at residue position 114 of the NS5 protein, a point-mutation from GTG to ATG at genomic position 8007 was introduced into ZIKV-Natal CPER fragments five and six using mutagenesis primers (Fig 2A and S1 Table). The V114M mutant virus (called M114 virus from herein) was recovered by transfecting the CPER product onto Vero76 cells. The viral supernatant from transfected cells were harvested at 6 days post-transfection (dpt) and clarified using low-speed centrifugation at 1000 g for 5 min at room temperature (rt). After determining the virus titre, the M114 mutant were further propagated in C6/36 cells at a multiplicity of infection (MOI) of 0.01 to generate passage 1 (p1) stocks used in this study. An 1826 bp amplicon region containing the mutagenesis site was generated from cDNAs of recovered M114 mutant virus stocks to authenticate its identity prior to experimentation by Sanger sequencing (Fig 2A).

### Virus titration

ZIKV titers were determined by immuno-plaque assay (iPA) on Vero76 cells as previously described [17], with following modification. A diluted (1:2000) primary anti-flavivirus envelope (E) protein humanized monoclonal antibody (mAb) 4G2 and diluted (1:2000) secondary goat anti-human antibody (Goat anti-human IRDye 800CW, LI-COR) was added at 50 μL per well to detect ZIKV in mice sera. All incubations were performed at rt for 1 h followed by 3 washes with 1× phosphate-buffered saline (PBS) Tween20 (0.1%). Virus titers were calculated based on respective dilution factors and quantified as focus-forming-units per mL (FFU/mL).

### *In vitro* infection with ZIKV

For viral growth kinetics assay, mammalian cells were seeded at 4 × 10^5^ cells per well, while mosquito cells were seeded at 1 × 10^6^ cells per well in six-well plates 12 h prior to experiment. Cells were then infected with C6/36-propagated stocks of V114 or M114 viruses at the indicated multiplicity of infection (MOI) in the figures. Briefly, 200 μL of respective virus inoculum at the required MOI was added to each well at either 37°C with 5% CO_2_ (mammalian cells), or 28°C (mosquito cells) with 5% CO_2_ for 1 h with 15 min interval rocking. After virus adsorption, the cell monolayers were washed thrice with additive-free DMEM/RPMI and replaced with 2.5 mL of media per well. At the timepoints after infection indicated in the figures, 120 μL of culture supernatant was collected from each sample wells and stored at -80°C until sample titration by iPA on Vero76 cells as described above.

### Quantitative reverse-transcription PCR (qRT-PCR)

Vero cells were infected with V114 or M114 ZIKV at MOI of 0.1. At 0, 24, 48 and 72 hours post-infection (hpi) cells were lysed and RNA was isolated using TRI reagent (Sigma) according to the manufacturer’s recommendations. Viral RNA quantification by qRT-PCR was performed using standard curve method as described previously [31] using the primers ZIKV-Natal_F and ZIKV-Natal_R (S1 Table).

### Immunoblot analysis

Cells were either seeded at 8 × 10^5^ cells per well 12 h prior to experiment and infected with respective viruses at indicated MOI. Thereafter, infected cells were washed thrice with additive-free DMEM and maintained in culture media for 24 h (mammalian) until sample collection. For analysis of phosphorylated STAT2 protein, cells were first treated with 10^3^ IU/mL recombinant human IFNα2 (R&D Systems) for 30 min at 37°C with 5% CO_2_ prior to sample collection (S1 Table). Total STAT2, phosphorylated STAT2, ZIKV-NS5, ZIKV-NS3 and glyceraldehyde-3-phosphate dehydrogenase (GAPDH) proteins were analyzed by western blot with the following antibodies at respective dilutions: rabbit anti-STAT2 and anti-phosphorylated-STAT2 (pSTAT2) (1:1000); rabbit anti-ZIKV-NS5 and anti-ZIKV-NS3 (1:2000); mouse anti-GAPDH (1:20,000); goat anti-rabbit/mouse secondary horseradish peroxidase (HRP) -conjugates (1:10,000) (S1 Table). Membranes were developed using SuperSignal West Pico PLUS Chemiluminescent Substrate (Thermo-Scientific) and visualized on Amersham Imager 600 system (GE Life Sciences).

### Mouse infection

Five to six-week-old *Ifnar*^−/−^ C57BL/6 mice were gender-balanced to a 1:1 male-to-female ratio and age-matched within the timeframe of one week prior to infection. Mice were then challenged with either V114 or M114 ZIKV (n = 10/group) at 1 × 10^5^ FFU per mice via intraperitoneal (i.p.) injection. Uninfected mice (n = 9) were set aside as naïve group, and viraemia from sera was monitored daily for 7 days post-infection. Sera was separated by centrifugation at 10,000 g for 10 min at 4°C. Clinical symptoms, weight changes and survival rate were measured for 21 days post-challenge [32]. To determine the severity of clinical and neurological symptoms, mice were assigned a score based on the following criteria [30]: 0 –no disease, 1 –hindlimb weakness or disrupting righting reflex, 2 –partial hindlimb paralysis or toe knuckling, 3 –complete paralysis of one hindlimb, 4 –complete paralysis of both hindlimbs, 5 –complete paralysis of all four limbs, moribund or dead. Mice presented with a score of 4 or 5 were euthanized. Two independent experiments were conducted.

### Mosquito infection

*Ae*. *aegypti* colony was established at the insectary facilities of the QIMR Berghofer Medical Research Institute from the eggs of *Wolbachia*-free adult females collected at Innisfail, Australia, in April 2016. Eggs were hatched and mosquito larvae were reared at densities of 300 larvae in 3 L rainwater and fed grounded TetraMin tropical fish food (Tetra, Melle, Germany) *ad libitum*. Three-five days old *Ae*. *aegypti* mosquitoes were fed blood meals containing a 1:1 mixture of either V114 or M114 ZIKV strains (3.8 × 10^8^ FFU/mL) and defibrinated sheep blood (Serum Australis) via artificial membrane feeders using procedures described previously [33]. Fed mosquitoes were collected and maintained in an environmental chamber (FitoClima 600 PLH, Aralab, Lisbon, Portugal) for 7 or 14 days at 28°C, 75% humidity and 12:12 h day:night light cycling. The mosquitoes were then anesthetized with CO_2_ and ice and legs and wings were removed and stored at -80°C. Saliva was collected from the immobilized mosquitoes by placing a capillary tube containing 10 μL of a solution containing 10% sucrose and 10% FBS [34] over the proboscis for 20 min. The solutions were then expelled into 1.5 mL microfuge tubes and stored at -80°C. Bodies were then collected and stored at -80°C. Mosquito bodies and legs and wings samples were homogenized using a Qiagen Tissuelyser II (Qiagen, Hilden, Germany) and centrifuged at 10,000 g for 10 min at 4°C. ZIKV titers in supernatants of body, legs and wings and saliva samples were determined by iPA as described above.

### Statistics

All statistics analyses were performed using GraphPad Prism 9.0.1 software (La Jolla, CA, USA). For *in vitro* virus growth kinetics and RNA replication, statistical differences were determined using two-way analysis of variance (ANOVA) with Tukey’s or Fisher’s LSD multiple comparison tests for pairwise comparison of datasets. For *in vivo* experiments, statistical differences were determined using Mann–Whitney U-test for non-parametric datasets with difference in variances of < 4 or if the difference of variances was > 4, the Kolmogorov–Smirnov test was employed, unless otherwise stated in the respective figure legends. The statistical difference for all experiments was set at *p* value ≤ 0.05.

## Results

### NS5-M114V mutation has no effect on virus replication in cells

Following recovery of the V114M reverse mutant virus (henceforth termed M114) and propagation of passage 1 working stock in C6/36 cells, the virus stocks were verified by Sanger sequencing prior to experiments to ensure retention of the mutation (Fig 2A). We examined the morphology of virus-induced foci and growth kinetics of ZIKV-M114 and wildtype ZIKV-Natal strain (henceforth termed V114) in different cells following infection at a multiplicity of infection (MOI) of 0.1 (Fig 2B and 2C). Foci morphology between the two viruses at 48 hours post-infection (hpi) was similar across all cell lines (Fig 2B). The growth kinetics of V114 and M114 viruses showed similar replication efficiencies in both vertebrate and invertebrate cell lines, regardless of whether the cells are competent in antiviral responses (A549, Aag2) or deficient in antiviral response pathways (Vero76 –IFNβ production deficient, *Ifnar*^-/-^ MEF–IFNα/β receptor deficient, and C6/36 –dicer-deficient) (Fig 2C). To test the potential effect of M114 mutation on RNA replication, viral RNA in infected Vero cells was assessed by qRT-PCR. No difference in viral RNA accumulation was detected between M114 and V114 viruses (Fig 2D). Overall, the results showed no significant differences between M114 and V114 viruses in replication in a panel of cell lines tested.

### NS5-M114V mutation has no effect on STAT2 degradation

**Fig 3:**
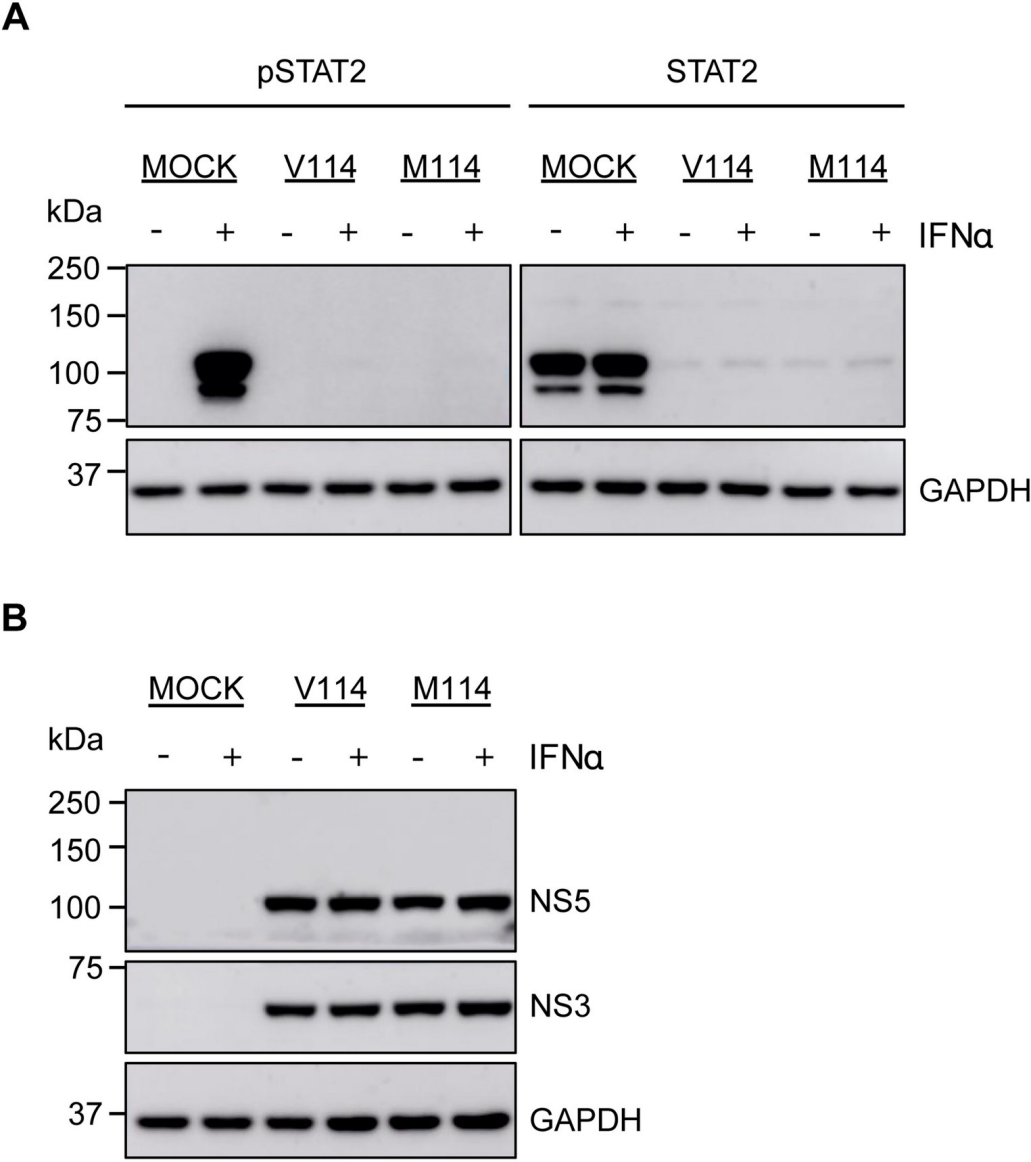
NS5-M114V mutation does not affect the ability of ZIKV to degrade STAT2 or inhibit its phosphorylation. Vero76 cells were infected with either V114 or M114 ZIKV strains at MOI 5.0 unless otherwise stated. At 24 hours post-infection (hpi), half of the respective infected cells were treated with human IFNα2 (10^3^ IU/mL) for 30 min prior to harvesting cell lysates. Harvested cell lysates were then analyzed by Western blot with relevant antibodies. (**A**) STAT2 phosphorylation (pSTAT2) and total STAT2 levels, (**B**) viral protein NS5 and NS3 levels in cells infected with V114 and M114 mutant viruses with or without IFNα2 treatment. The GAPDH levels acted as a loading control. One representative immunoblot from three independent experiments is shown.

As ZIKV-NS5 antagonizes type I interferon (IFN) signaling by facilitating STAT2 degradation [35,36], we sought to assess if NS5-M114V mutation could affect the degradation of STAT2. Vero cells were used for analyzing viral suppression of type I IFN signaling as they do not produce IFNβ upon viral infection but retain a robust response to exogenous IFNα/β treatment [37,38]. Vero cells were infected with either M114 or V114 viruses at MOI 5.0 and at 24 hours post-infection (hpi), cells were either exposed to 10^3^ IU/mL recombinant human IFNα2 for 30 mins or left unexposed. Both, total STAT2 levels and the levels of phosphorylated STAT2, were then determined in infected cell lysates by immunoblot analysis with corresponding antibodies. The results showed barely detectable levels of total STAT2 and no phosphorylated STAT2 for both M114 and V114 viruses (Fig 3A), indicating that the M114V mutation in NS5 protein did not alter its ability to degrade STAT2 or affect STAT2 phosphorylation. Levels of NS5 and NS3 viral proteins remained similar in cells infected with either virus (Fig 3B) demonstrating similar virus replication efficiencies and NS5 expression. Altogether, the results demonstrated that the M114V mutation in NS5 had no effect on the virus’ ability to degrade STAT2 or inhibits its phosphorylation.

### NS5-M114V mutation does not affect overall virus virulence in *Ifnar*^-/-^ mice

**Fig 4:**
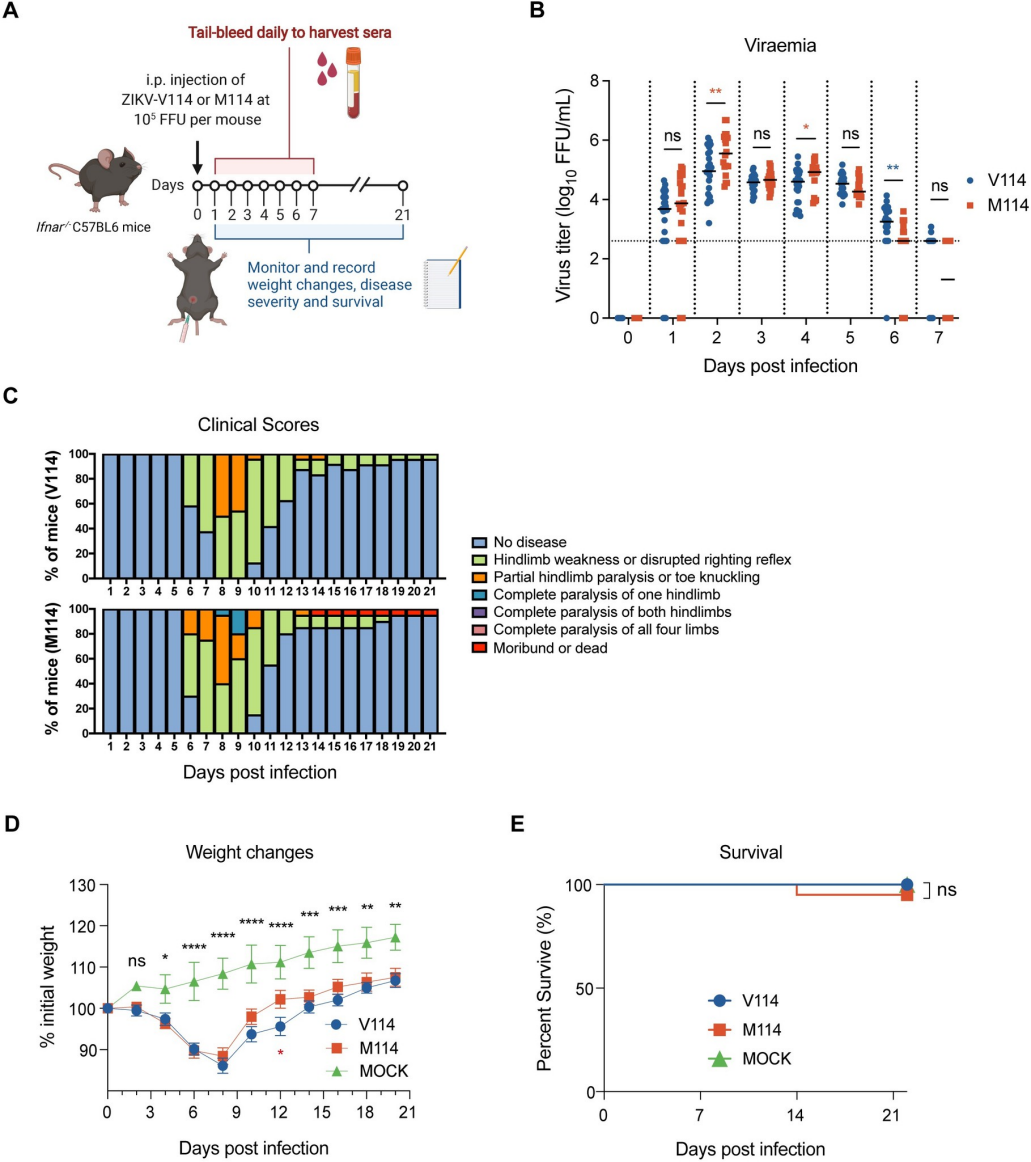
Characterization of ZIKV NS5-M114V mutation on virulence in mice. (**A**) Schematic outline of mice study created with BioRender.com. Five-to-six weeks-old gender-balanced *Ifnar*^-/-^ mice were either uninfected (naïve) or intraperitoneally (i.p.) challenged with 10^5^ focus-forming-units (FFU) of NS5-V114 or NS5-M114 ZIKV (10 mice/group) and monitored for 21 days. Two independent experiments were performed, and combined data from two experiments were presented. (**B**) Virus titers from sera harvested daily for 7 days post infection and determined by immuno-plaque assay (iPA). Horizontal dotted lines represent the limit of detection of 2.6 log_10_ FFU/mL. (**C**) Clinical scores for disease symptoms and progression monitored daily for 21 days. (**D**) Weight changes of mice measured every 2 days upon infection for 21 days. (**E**) Overall survival rate of V114 and M114 ZIKV infected mice compared to naïve uninfected mice over 21 days. The statistical significance of differences in viremia, weight changes and Kaplan-Meier survival curves between strains was determined using Mann-Whitney test and Log-rank (Mantel-Cox) test respectively. “ns”, not significant, *p ≤ 0.05, **p ≤ 0.005, ***p ≤ 0.0005, ****p ≤ 0.0001.

Next, we sought to determine whether the M114V mutation affects virulence *in vivo* [30]. Five- to six-week-old *Ifnar*^-/-^ C57BL/6 mice were challenged with 10,000 focus-forming-units (FFU) of M114 or V114M viruses per mice via intraperitoneal injection and were tail-bled for viremia (daily for the first 7 days), monitored for weight changes (every 2 days), disease severity (clinical scores; daily) and survival (every 2 days) over a course of 21 days post-infection (Fig 4A). From day 1 to day 4 post-infection, ZIKV titers in sera samples from M114-infected mice slightly exceeded V114-infected mice at 2 and 4 days post-infection (Fig 4B). At later time points (5 and 6 days post-infection), virus titers in V114-infected mice slightly exceeded those in mice infected with M114 (Fig 4B). From 6 days post-infection, half of the M114-infected mice displayed earlier onset of severe neurological impairments such as complete paralysis of one hindlimb compared to V114-infected mice, which exhibited partial hindlimb paralysis and toe knuckling (Fig 4C). Neurological impairments persisted in about 15% of all infected mice regardless of virus strains until the end of the experiment (Fig 4C). All ZIKV-infected mice began losing weight from 4 days post-infection, and by days 6 to 8, all mice had lost between 10% to 15% of their starting body weight (Fig 4D). In tandem with the reduction in viremia at 7 days post-infection, all infected mice began recovering from weight loss at 8 days post-infection (Fig 4D). All V114-infected mice survived the challenge while one of 10 mice infected with M114 succumbed to infection (Fig 4E). Overall, the results indicated that the M114V mutation did not have a substantial effect on overall virus virulence but slightly reduced disease symptoms and slightly prolonged viraemia.

### NS5-M114V mutation reduced infection and dissemination rates in *Aedes aegypti* mosquitoes but did not affect virus secretion into saliva

As the main transmission vector of ZIKV [39], *Ae*. *aegypti* is distributed widely across both Southeast Asia and Latin-America [40], we sought to determine whether the NS5-M114V mutation might affect mosquito infection (bodies), dissemination (legs and wings) and transmission (saliva) in *Ae*. *aegypti*. Groups of 50 *Ae*. *aegypti* mosquitoes were fed with defibrinated sheep blood (Serum Australis, New South Wales, Australia) containing 3.8 × 10^8^ FFU/mL of M114 or V114 viruses. Respective bodies, legs and wings, and saliva were collected at 7 and 14 days post-infection for virus titration.

**Fig 5:**
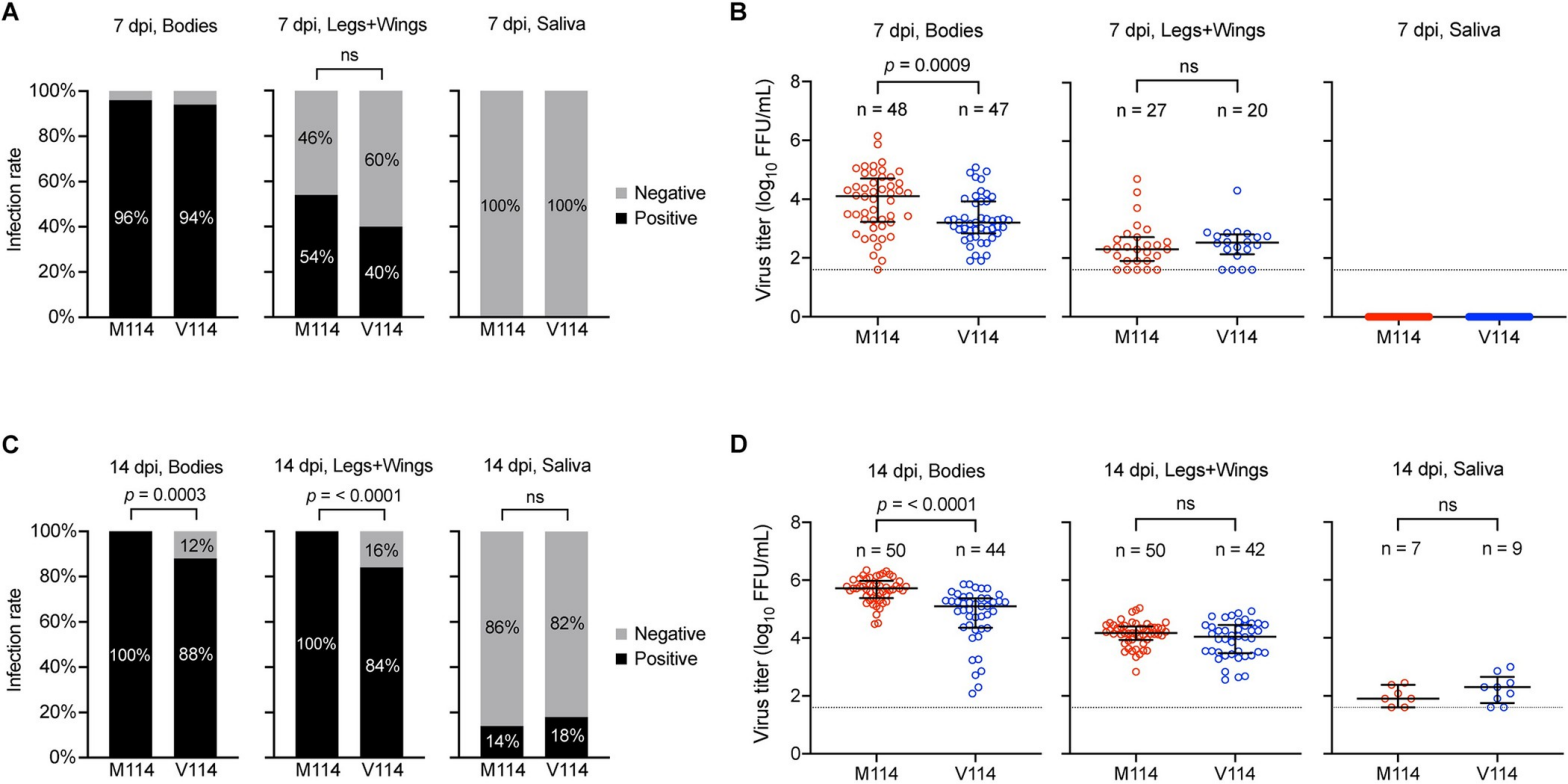
The ZIKV NS5-M114V mutation reduced infection and dissemination rates in Aedes aegypti mosquitoes but had no effect on virus secretion into the saliva. Female *Ae*. *aegypti* mosquitoes were exposed to infectious blood meals containing respective ZIKV strains at 10^8^ FFU/mL. Infected mosquitoes’ bodies, legs and wings, saliva expectorates (n = 50/group) were harvested at (**A**, **B**) 7 days post-infection (dpi) and (**C**, **D**) 14 dpi for infectious virus particle titration using immuno-plaque assay (iPA). To determine the infection rate of each strain at 7 dpi (**A**) and 14 dpi (**C**), the number of respective ZIKV-positive bodies, legs and wings, and saliva expectorates were measured and expressed as percentages for comparison. To determine the efficiency of virus replication, dissemination and transmission potential, viral titers from ZIKV-positive bodies, legs and wings, and saliva expectorates at 7 dpi (**B**) and 14 dpi (**D**) were quantified and expressed as log_10_ transformed FFU/mL. Horizontal lines indicate the median and interquartile ranges of individual viral titer values and dotted lines indicate the limit of detection of the assay (1.6 log_10_ FFU/mL). Statistical significance was determined using chi-squared (**A**, **C**) or Mann-Whitney U tests (**B**, **D**). All *p*-values indicated are two-sided, with no multiple comparisons performed in each test.

At 7 days post-infection, V114 and M114 viruses were detected in 94% and 96% of the bodies, respectively, and in 40% and 54% of the legs and wings, respectively (Fig 5A). No virus was detected in the saliva expectorates for both viruses at 7 days post-infection (Fig 5A). Virus titers were higher in bodies of M114-infected mosquitoes compared to V114 (*p* = 0.0009), but no differences were observed between both viruses in the legs and wings (Fig 5B). By 14 days post-infection, virus was detected in all mosquito samples (bodies, legs and wings, saliva) for both viruses. Infection rates in the bodies, or legs and wings were lower for V114-infected mosquitoes compared to M114-infected mosquitoes (88% and 84%, respectively for V114 or 100% for M114) (Fig 5C). In contrast, the infection rates in the saliva were not significantly different between the two viruses at 18% and 14%, respectively (Fig 5C). Virus titers in bodies, legs and wings, and saliva expectorates for both viruses at 14 days post-infection were increased compared to those at 7 days post-infection. Virus titers in M114-infected bodies, legs and wings were higher compared to V114-infected mosquitoes (Fig 5D). However, no significant differences were observed between virus titers in saliva expectorates at 14 days post-infection (Fig 5D). Collectively, the results suggest that the NS5-M114V mutation had no effect on virus secretion into the saliva.

## Discussion

Here, we addressed the potential effect of NS5-M114V substitution fixed in American ZIKV isolates on various virus properties with a goal of understanding whether it could potentially contribute to the unprecedented outbreak in the Americas. We demonstrated that the NS5-M114V mutation had no effect on virus replication in different cell lines, the ability of ZIKV to degrade STAT2, overall virulence in *Ifnar*^-/-^ mice, or virus secretion into saliva of *Ae*. *Aegypti* mosquitoes. The NS5-M114V mutation, however, slightly prolonged viremia in *Ifnar*^-/-^ mice but lowered ZIKV infection and dissemination rates in mosquitoes.

Earlier works have demonstrated that emerging mutations such as prM-S17N and NS1-A188V enhance ZIKV virulence and transmissibility among American ZIKV isolates [15,19]. This suggests a potential contribution to the differences in outbreak magnitude observed between outbreaks in the Pacific Islands and Asia from 2007–2016 and those that occurred in the Americas during 2015–2016 [16,18]. Among other identified mutations, the NS5-M114V mutation is of particular interest as it is present in all American ZIKV isolates but not in isolates from the Pacific Islands and Southeast Asia and was therefore hypothesized to influence the epidemic outcomes of ZIKV [21–23,41].

The findings reported in this present study are consistent with other studies on the potential impact of NS5-M114V mutation. Previously, Zhao *et al* reported negligible impact of this mutation on virus replication in cells and *in vivo* virulence in AG6 mice (which lack both type I and type II IFN receptors), using another American ZIKV isolate (V114) and its corresponding M114 mutant virus [28]. Our findings agreed with this study such that mutations at position 114 (either M➔V or V➔M) have no impact on virus replication in cells. However, unlike the findings from Zhao *et al*, we observed some difference to the duration of viremia between V114 and M114 viruses in *Ifnar*^-/-^ mice. We hypothesize that the differences observed in our study might be attributed to the different immunodeficiency status of the mouse models used; i.e., the presence or absence of type II IFN signaling.

Type II IFN (IFNγ) are key inducers of proinflammatory cytokines such as IRF1, IFIT1, CXCL10 [42] and crucially, known cellular receptors for ZIKV infection such as AXL, Tyro3, and DC-SIGN [43]. Since ZIKV was demonstrated to promote IFNγ activation by increasing the presence of STAT1-STAT1 homodimers through a reduction of STAT2 levels via NS5-mediated proteasomal degradation [44,45], this would lead to an increased expression of both proinflammatory cytokines and ZIKV cellular entry factors which may contribute to ZIKV dissemination in *Ifnar*^-*/-*^ mice but not in AG6 mice [46]. Consistent with previous reports on the proteasomal degradation of STAT2 via an unknown E3 ubiquitin-ligase facilitated by ZIKV-NS5 [35], we demonstrated that ZIKV efficiently degrades STAT2 and that NS5-M114V mutation does not impact the ability of ZIKV to degrade STAT2. However, given that prolonged viremia duration was observed in V114 infected *Ifnar*^*-/-*^ mice, there could be a potential effect of NS5-M114V substitution on IFNγ signaling which remains to be explored in future studies.

Infection studies in *Ae*. *Aegypti* mosquitoes showed small but statistically significant decrease in the infection and dissemination rates, as well as decrease in viral titers in the bodies, legs and wings for the V114 virus compared to the M114 virus. This indicates a decreased potential for the V114 virus to establish infection in mosquitoes, contrary to our hypothesis. Similarly, we did not find significant differences in the viral titers in the saliva expectorates, indicating that the NS5-M114V mutation is unlikely to affect transmissibility of ZIKV. This also suggests that the establishment of viral replication in salivary glands and/or virus secretion into saliva may not be directly dependent on the efficiency of viral replication in the midgut.

An important principal limitation of our study is that in the absence of positive evidence supporting an alternative hypothesis, we cannot simply accept the null hypothesis. Therefore, we are unable to completely refute the potential role of NS5-M114V mutation on the differences in outbreak magnitude between Southeast Asia and the Americas, based solely on our negative findings. However, combined with the published data, our findings reinforce the notion that mutations acquired after 2013 by ZIKV are unlikely to influence the epidemic potential of ZIKV [20]. Recent publications have demonstrated that adaptive mutations in Asian strains acquired by ZIKV starting from 2007 and prior to 2013 that represented “reversions” to the residues in an earlier African strain (C-T106A, prM-V1A, E-V473M, NS1-A188V and NS5-M872V) improved ZIKV transmission efficiency by *Ae*. *aegypti* as well as enhanced infection and viremia in nonhuman primates and mouse models [16,18,20]. These mutations therefore were implied to contribute to emergence of ZIKV in the Pacific Islands and Americas. On the other hand, Shan *et al* [16] reported insignificant impacts of NS5-M114V mutation (acquired during virus migration to Americas) on ZIKV neurovirulence using CD1 mice and Zhao *et al* [28] also didn’t find significant effects of NS5-M114V mutation on various virus properties as discussed above. Additionally, despite NS5-M114V mutation not being present in Asian ZIKV isolates circulating during 2015–2016, large outbreaks like the one in Singapore were still recorded [24,47]. Interestingly, as Liu *et al* pointed out [20], the Singaporean ZIKV isolates were distinct from other Asian lineages, and importantly contained the adaptive mutations/reversions mentioned above, including the C-T106A which showed the strongest effect on virus fitness. Taken together, these findings and our results suggest that the NS5-M114V mutation, introduced only in 2015, is unlikely to contribute to the epidemic outcomes of contemporary ZIKV strains, and that the epidemic potential of the virus responsible for the 2015/2016 ZIKV outbreak was likely locked in place prior to 2013.

Another caveat of our study is that we did not examine whether the NS5-M114V mutation affects ZIKV’s capability to cause CZS. Previously, we have investigated the determinants of CZS using pregnant mouse model and have consistently observed transplacental infection of fetal brains by representatives of different ZIKV lineages (African–MR766 strain; Asian [Malaysian]– P6-740 strain; and American–Natal-RGN strain), despite each having different residues (M/T/V, respectively) at position 114 in NS5 [29,48]. Therefore, residues at this position are unlikely to have any influence on CZS. While CZS is indeed an important public health problem associated with ZIKV infection, it is neither a de-facto consequence of ZIKV infection in humans nor a prerequisite for ZIKV transmission cycle. In fact, approximately 18% of laboratory-confirmed human ZIKV infections reported self-limiting flu-like symptoms such as fever, malaise, and maculopapular rashes [7,49,50], while incidences of CZS are reported only in ~4.5–6% of confirmed ZIKV-positive pregnancies cases [51,52]. Hence, we believe that the bigger public health concern is the potential to cause explosive outbreaks by contemporary ZIKV strains as witnessed during the 2015–2016 Latin-America outbreak. Therefore, the rationale of our study focused on investigating whether the NS5-M114V mutation, which is only present in all contemporary American ZIKV isolates but not in contemporary Asian isolates or pre-2015 ZIKV isolates, could affect virus properties and transmission that would contribute to the magnitude of ZIKV outbreak observed in the Americas.

In conclusion, while not without limitations, our findings complement findings from several other studies and altogether show that NS5-M114V mutation, which occurred during the introduction of ZIKV into the Americas, is unlikely to be responsible for elevating the epidemic potential of American ZIKV isolates at that point. Additionally, our data provide extensive characterization of NS5-M114V mutation in mammalian and mosquito hosts and suggest that not all mutations identified in emerging viral isolates may result in a fitness advantage.

## Supporting information

S1 Table Key resources used in this study. (PDF)
