# Supplementary material for: The distinguishing NS5-M114V mutation in American Zika virus isolates has negligible impacts on virus replication and transmission potential
Source: PLoS Negl Trop Dis. 2022 May 10;16(5):e0010426. doi: 10.1371/journal.pntd.0010426 (PMC9122223; doi:10.1371/journal.pntd.0010426)
Supplement: S1 Table — (PDF) [file pntd.0010426.s001.pdf]

Supporting information

S1 Table. Key resources used in this study.

Reagents/Resources	Source	Identifier
Antibodies		
Mouse monoclonal anti-Flavivirus Envelope clone 4G2	Kindly provided by Prof. Roy A. Hall	N/A
Humanized monoclonal anti-Flavivirus Envelope clone 4G2	Kindly provided by A/Prof. Daniel Watterson	N/A
Rabbit polyclonal anti-ZIKV NS5	Kindly provided by Prof. Andres Merits	N/A
Rabbit polyclonal anti-Stat1α (C-24)	Santa Cruz	sc-345 (Discontinued)
Rabbit monoclonal anti-pStat1 (58D6)	Cell Signaling Technology	Cat# 9167
Rabbit monoclonal anti-Stat2 (D9J7L)	Cell Signaling Technology	Cat# 72604
Rabbit monoclonal anti-pStat2	Cell Signaling Technology	Cat# 88410
Mouse monoclonal anti-GAPDH	Sigma	Cat# G8795
Goat anti-mouse IgG IRDye 800CW	LI-COR	P/N: 926-32210
Goat anti-human IgG IRDye 800CW	LI-COR	P/N: 926-32232
Goat anti-Mouse IgG-HRP conjugate	Cell Signaling Technology	Cat# 7076S
Goat anti-Rabbit IgG-HRP conjugate	Cell Signaling Technology	Cat# 7074S
Virus Strain		
ZIKV strain Natal-RGN 2015	Setoh et al., 2017. mSphere.	GenBank accession number: KU527068.1
Enzymes, Peptides, and Recombinant Proteins		
Human IFNα2	R&D Systems	Cat# 11101-1
SuperScript IV Reverse Transcriptase	Invitrogen	Cat# 18090010
PrimeSTAR GXL DNA Polymerase	Takara Bio	Cat# R050A

Critical Commercial Assays & Kits		
Lipofectamine LTX Reagent with PLUS Reagent	Invitrogen	Cat# 15338030
Monarch DNA Gel Extraction Kit	New England Biolabs	Cat# T1020L
20× Bolt MES SDS Running Buffer	Invitrogen	Cat# B0002
Bolt 4–12% Bis-Tris 15-well Mini Protein Gel	Invitrogen	Cat# NW04125BOX
Precision Plus Protein Kaleidoscope Pre-Stained Protein Standards	Bio-Rad	Cat# 1610375
SuperSignal West Pico PLUS Chemiluminescent Substrate	Thermo Scientific	Cat# 34577
Pierce Clear Milk Blocking Buffer (10×)	Thermo Scientific	Cat# 37587
Cell Lines		
Human: A549 cells	ATCC	Cat# CCL-185
Ae. albopictus: C6/36 cells	ATCC	Cat# CRL-3216
Ae. aeygpti: Aag2 cells	N/A	RRID: CVCL_Z617
Monkey: Vero76 cells	ATCC	Cat# CRL-1587
Mouse: *Ifnar*$^{-/-}$ MEF cells	Melian et al., 2013. J Gen Virol	N/A
Oligonucleotides (5' – 3')		
V114M-Forward (F) CATGAAGAACCCATGTT GGTGCAAAG	This study	N/A
V114M-Reverse (R) CTTTGCACCAACATGGG TTCTTCATG	This study	N/A
V114M-Sanger_F ACGCTGGCTTGGTCAAG AGA	This study	N/A
V114M-Sanger_R CGGATCCTTTCAATGCG GTTACCAAT	This study	N/A
ZIKV-Natal_F GAGTGTGATCCAGCCGT TATT	Slonchak et al., 2020. Nat Commun	N/A
ZIKV-Natal_R CAGCCTCCATGTGTCATT CT	Slonchak et al., 2020. Nat Commun	N/A

Software and Algorithms	
GraphPad Prism version 9.0.1	GraphPad
ImageJ version 1.53g	NIH
CLC Main Workbench 7.6.2	Qiagen
Adobe Illustrator version 25.1	Adobe
Image Studio version 4.0	LI-COR
